# Comparison of the cox regression to machine learning in predicting the survival of anaplastic thyroid carcinoma

**DOI:** 10.1186/s12902-023-01368-5

**Published:** 2023-06-05

**Authors:** Lizhen Xu, Liangchun Cai, Zheng Zhu, Gang Chen

**Affiliations:** 1 Shengli Clinical Medical College of Fujian Medical University, 350001 Fuzhou, China; 2 Department of Endocrinology, Fujian Provincial Hospital, 350000 Fuzhou, China; 3 Fujian Provincial Key Laboratory of Medical Analysis, Fujian Academy of Medical Sciences, Fuzhou, China

**Keywords:** Anaplastic thyroid carcinoma, SEER program, Survival analysis, Cox regression, Machine learning, SHAP

## Abstract

**Background:**

To compare the ability of the Cox regression and machine learning algorithms to predict the survival of patients with Anaplastic thyroid carcinoma (ATC).

**Methods:**

Patients diagnosed with ATC were extracted from the Surveillance, Epidemiology, and End Results database. The outcomes were overall survival (OS) and cancer-specific survival (CSS), divided into: (1) binary data: survival or not at 6 months and 1 year; (2): time-to-event data. The Cox regression method and machine learnings were used to construct models. Model performance was evaluated using the concordance index (C-index), brier score and calibration curves. The SHapley Additive exPlanations (SHAP) method was deployed to interpret the results of machine learning models.

**Results:**

For binary outcomes, the Logistic algorithm performed best in the prediction of 6-month OS, 12-month OS, 6-month CSS, and 12-month CSS (C-index = 0.790, 0.811, 0.775, 0.768). For time-event outcomes, traditional Cox regression exhibited good performances (OS: C-index = 0.713; CSS: C-index = 0.712). The DeepSurv algorithm performed the best in the training set (OS: C-index = 0.945; CSS: C-index = 0.834) but performs poorly in the verification set (OS: C-index = 0.658; CSS: C-index = 0.676). The brier score and calibration curve showed favorable consistency between the predicted and actual survival. The SHAP values was deployed to explain the best machine learning prediction model.

**Conclusions:**

Cox regression and machine learning models combined with the SHAP method can predict the prognosis of ATC patients in clinical practice. However, due to the small sample size and lack of external validation, our findings should be interpreted with caution.

**Supplementary Information:**

The online version contains supplementary material available at 10.1186/s12902-023-01368-5.

## Introduction

Thyroid carcinoma is the fifth most common cancer among women in the United States [1]. In recent decades, the incidence of thyroid carcinoma has increased dramatically in many countries [2]. A study of the United States analyzed the 10-year data from 2007 to 2016, and reported that the incidence of thyroid carcinoma among young people of all ages (15–39 years old) ranked the top three [3]. Among thyroid carcinoma, anaplastic thyroid carcinoma (ATC) accounts for 1-2% [4], but it is the most aggressive type and highly malignant, which is the main cause of death associated with thyroid malignant tumors. The median survival time of ATC is only 5–6 months [5]. The quality of life among ATC patients is significantly reduced, coupled with persistent occupation of medical resources and high mortality rate, which result in a heavy economic and social burden. Therefore, accurate prediction of ATC patient survival and understanding the drivers of these predictions are critical for clinically targeted therapy. 

The known risk factors related to the prognosis of ATC include age, sex, race, marital status, insurance, socioeconomic status, level of education, tumor stage, tumor size, multifocality, surgery, radiotherapy, chemotherapy and so on [6–11]. Additionally, the AJCC 8th edition reveals a better performance than the AJCC 7th edition TNM staging in predicting survival of ATC patients [12]. Traditional methods for predicting survival of ATC patients are based on existing clinical and sociodemographic predictors, using Cox proportional hazards (Cox) regression analysis to establish nomograms [12–16]. Although the estimated C-index calculated by some models appears to be ideal, there is still a risk of overfitting. With the rapid development of precision medicine, machine learning (ML) has been widely applied in medical fields such as outcome prediction, diagnosis, medical image interpretation and treatment [17]. Applications of ML in thyroid carcinoma consist of diagnosis, nodule identification and risk factor analysis [18–21]. However, rare data show applications of ML for prognostic analysis in ATC patients. ML does not need to assume the relations between input variables and outputs variables, as well as takes into account all possible interactions and effect corrections between variables [22]. More importantly, ML is an efficient and accurate substitute of semi-parametric and parametric models.

In this study, we aimed to compare the application of Cox regression and ML algorithms for survival prediction among ATC patients. Strategies aimed at selecting most suitable predictive model could help clinicians to intervene risk factors timely and prescribe treatments properly, enhancing the understanding of decision-making process for assessing ATC.

## Methods

### Study population

The data for this study were obtained from the SEER database: Surveillance, Epidemiology, and End Results (SEER) Program (www.seer.cancer.gov) SEER*Stat Database: Incidence - SEER 18 Regs Custom Data (with additional treatment fields). We used SEER*stat 8.3.9 software to obtain clinical data of patients diagnosed with anaplastic thyroid carcinoma during 2004–2015. The inclusion criteria were as follows: (1) topography code: C73.9 Thyroid gland; (2) ICD-O-3 histologic codes: 8020–8035, including “8020/3: Carcinoma, undifferentiated, NOS, 8021/3: Carcinoma, anaplastic, NOS, 8022/3: Pleomorphic carcinoma, 8030/3: Giant cell and spindle cell carcinoma, 8031/3: Giant cell carcinoma, 8032/3: Spindle cell carcinoma, NOS, 8033/3: Pseudosarcomatous carcinoma, 8034/3: Polygonal cell carcinoma, 8035/3: Carcinoma with osteoclast-like giant cells. We excluded patients with Not first malignant primary and Survival time unknown or less than 1 month.

### Predictor variables and outcomes

We collected all relevant data including Age, Sex, Race, Marital status, Insurance, No high school diploma, Families below poverty, AJCC TMN stage, Tumor size, Multifocality, Regional lymph node surgery, Thyroid surgery, Radiotherapy, Chemotherapy. According to the AJCC 8th edition TNM staging system for thyroid cancer, different TNM staging were converted into 8th edition TNM staging uniformly [23]. The T staging of ATC is not only classified in T4 stage. We use the same definition for T staging for ATC and differentiated thyroid cancer (DTC). According to tumor size and tumor extension, the different T staging of AJCC were unified into AJCC 8th edition T staging, which was divided into T1 stage, T2 stage, T3a stage, T3b stage and T4 stage. In this study, X-tile software was used to analyze continuous variables to obtain the best cut-off value and group them. The variables analyzed by X-tile software included: Age, No high school diploma, Families below poverty, Tumor size. In addition, groups with small numbers were merged: T1 and T2 stages of AJCC were merged into T1-2, and T3a and T3b stages were merged into T3 stage.

The primary endpoint of the study were overall survival (OS) and cancer-specific survival (CSS). OS was defined as the time interval from diagnosis to death from all causes, and CSS was from diagnosis to death from that tumor alone. According to the different types of outcomes, we divided them into binary outcomes: 6-month OS, 12-month OS, 6-month CSS and 12-month CSS. In addition, the outcomes were also divided into time-to-event data for analysis.

### Data preprocessing

We counted the missing rates of all predictors, and retained factors with a missing rate of less than 30%. K-Nearest Neighbor (KNN) algorithms were used to fill missing values. Multicollinearity is explained by the variance inflation factor (VIF). VIF < 10 indicates that there is no multicollinearity among the variables. Correlation was determined by Spearman correlation analysis. A correlation coefficient greater than 0.5 indicates a significant correlation between variables.

### Model development and evaluation

For binary outcomes, we used four machine learning algorithms, Logistic, Random Forests, Extreme Gradient Boosting (XGBoost) and Adaptive Boosting (AdaBoost), to construct models and compared the pros and cons of these models. Similarly, for time-event outcomes, we compared the models constructed by COX regression with five machine learning algorithms: Survival Tree, Survival Support Vector Machine (SVM), Random Survival Forests, XGBoost and DeepSurv. In this study, 70% of all patients were used for training and 30% for validation using random number table method. The differences between the training set and validation set depended on the type of outcome variable. If the outcome variable was continuous, t-test was used, while if the outcome variable was categorical, chi-square test or Fisher’s exact test was used. In Cox regression, we use the bidirectional stepwise regression method for variable screening which automatically screens the variables with the smallest Akaike information criterion (AIC) to construct the model. The results of the Cox regression model are presented in the nomogram. In machine learnings, we use the XGBoost method to filter variables. We use a combination of grid search and multiple cross-validation to select the parameter values corresponding to the best C-index values as model parameters [24].

In order to avoid overfitting, the evaluation of the model comprehensively considers the results of the training set and the validation set, but mainly the results of the validation set. We used the C-index to describe the discriminativeness of the model. The C-index value can generally judge the generalization ability of the model: 0.5–0.7 means that the model has a weak generalization ability, 0.7–0.85 moderate, and 0.85-1.0 strong. In addition, we also use multiple evaluation indicators such as accuracy, sensitivity, and specificity to comprehensively evaluate the discriminative ability of the machine learning model. We used the calibration curve and brier-score to evaluate the calibration of the model. In the calibration plot, the X-axis represents the predicted survival time and the Y-axis the actual survival time with the predicted rate falling on the 45° diagonal in a perfect prediction model. The lower the Brier-score value, the better the calibration. We assessed the net benefit of the model for clinical decision making through the DCA curve. Kaplan-Meier analysis and log-rank test were used to explore differences in survival between risk subgroups.

### Model interpretation

The SHapley Additive exPlanation (SHAP) is a unified framework for interpreting the results of machine learning models [25]. We utilized SHAP to provide explanations for the final model, including associated risk factors causing death in patients with ATC and the importance of sorting features. Our study was reported following the TRIPOD (Transparent Reporting of a multivariable prediction model for Individual Prognosis Or Diagnosis) statement [26]. All statistical analyses in this study were performed using R software (version 4.0.2) and Python software (version 3.7.6). P value of < 0.05 was considered statistically significant.

## Results

### Patient characteristics

**Fig. 1:**
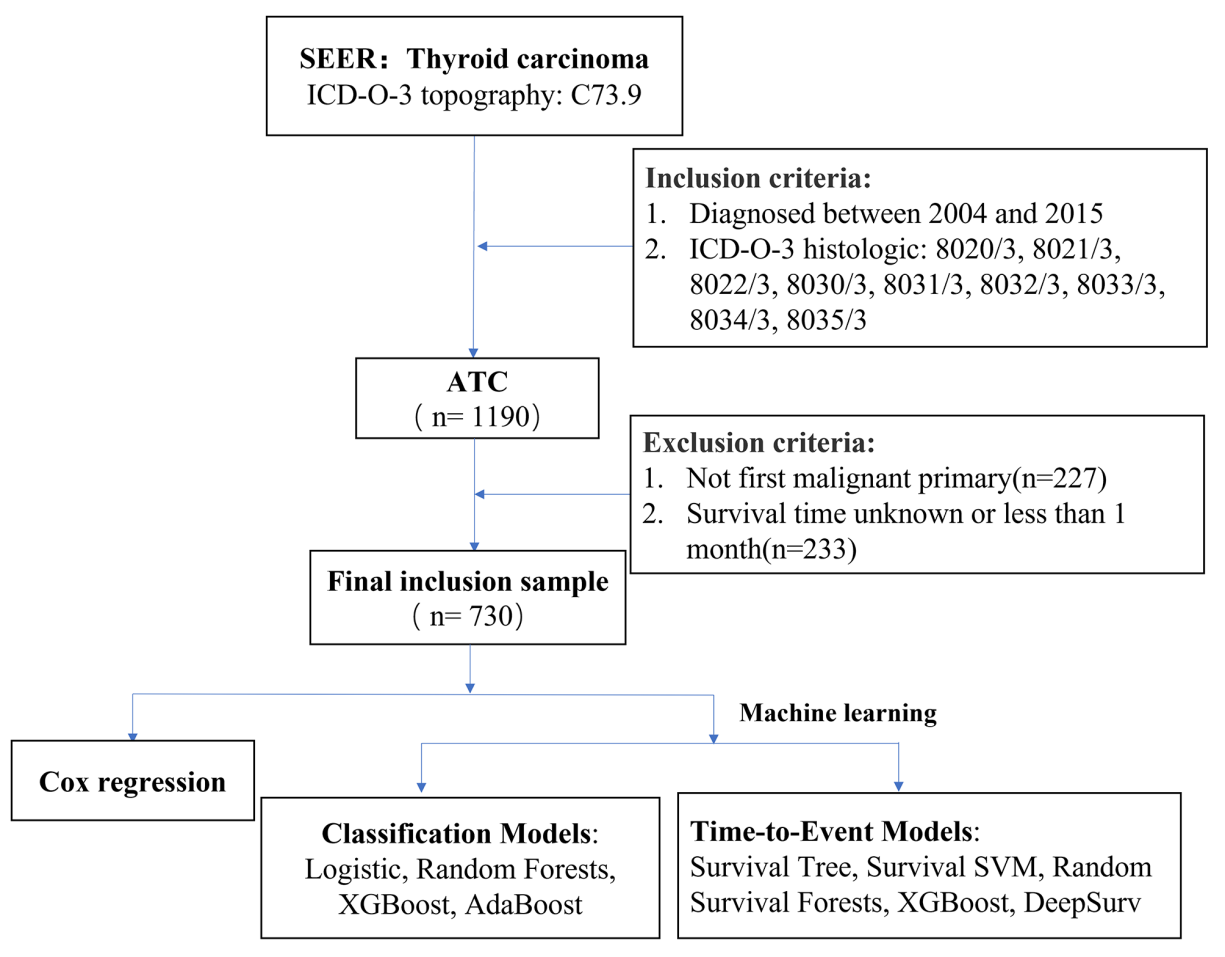
Flow chart of the screening of SEER database. (**Abbreviations**: **ATC**: anaplastic thyroid carcinoma; **XGBoost**: Extreme Gradient Boosting; **AdaBoost**: Adaptive Boosting; **SVM**: Support Vector Machine)

1190 patients diagnosed with ATC were identified from the SEER database from 2004 to 2015. According to the exclusion criteria, 730 patients were finally included. The flow-process diagram of data screening was shown in Fig. 1. Since the missing rate of each variable was < 30%, we did not remove the variable with low missing rate. The variables with the highest missing rate were insurance (22.9%), tumor size (19.5%) and multifocality (18.4%). According to the X-tile program, the optimal age cutoff points were 60 and 80 years old, and age groups were divided into < 60, 60–79 and ≥ 80 years. The optimal cutoff points divided tumor size into < 6 cm and ≥ 6 cm, no high school diploma into < 21% and ≥ 21%, and families below poverty into < 14% and ≥ 14%.

Table 1 shows Demographic characteristics of patients with ATC. 540 (74.0%) the elderly with age ≥ 60 years, 441 (60.4%) female and 580 (79.5%) the white race were the main cases. According to the 8th edition of the TNM staging system, stage IV accounted for the great majority of cases (610, 83.6%), followed by stage N1 (431, 59.0%) and stage M0 (400, 54.8%). 385 patients (52.7%) underwent surgical treatment, of which 138 (18.9%) underwent thyroid lobectomy and 247 (33.8%) underwent total or subtotal thyroidectomy. 281 patients (38.5%) received radiotherapy, and nearly half of patients (376, 51.5%) received chemotherapy. The 6-month and 12-month OS were 35.2% and 23.0%, respectively. The 6-month and 12-month CSS were 38.1% and 23.0%, respectively.

**Table 1:** Demographic characteristics of patients with ATC

	Overall (N = 730)	Trainingset (N = 510)	Testset (N = 220)	P-value
**Age**				
< 60	190 (26.0%)	142 (27.8%)	48 (21.8%)	0.527
60–79	155 (21.2%)	262 (51.4%)	123 (55.9%)	
≧ 80	385 (52.7%)	106 (20.8%)	49 (22.3%)	
**Sex**				
Female	441 (60.4%)	299 (58.6%)	142 (64.5%)	0.364
Male	289 (39.6%)	211 (41.4%)	78 (35.5%)	
**Race**				
White	580 (79.5%)	395 (77.5%)	185 (84.1%)	< 0.001
Black	65 (8.9%)	50 (9.8%)	15 (6.8%)	
Other	85 (11.6%)	65 (12.7%)	20 (9.1%)	
**Marital.status**				
Married	415 (56.8%)	286 (56.1%)	129 (58.6%)	0.612
Widowed	148 (20.3%)	105 (20.6%)	43 (19.5%)	
Single	103 (14.1%)	72 (14.1%)	31 (14.1%)	
Divorced or separated	64 (8.8%)	47 (9.2%)	17 (7.7%)	
**Insurance**				
Insured and any medical	714 (97.8%)	496 (97.3%)	218 (99.1%)	0.098
Uninsured	16 (2.2%)	14 (2.7%)	2 (0.9%)	
**No.high.school.diploma**				
< 21%	552 (75.6%)	378 (74.1%)	174 (79.1%)	0.006
≧ 21%	178 (24.4%)	132 (25.9%)	46 (20.9%)	
**Families.below.poverty**				
< 14%	492 (67.4%)	340 (66.7%)	152 (69.1%)	0.197
≧ 14%	238 (32.6%)	170 (33.3%)	68 (30.9%)	
**AJCC.T.8th**				
T1-2	29 (4.0%)	18 (3.5%)	11 (5.0%)	0.449
T3	91 (12.5%)	64 (12.5%)	27 (12.3%)	
T4	610 (83.6%)	428 (83.9%)	182 (82.7%)	
**AJCC.N.8th**				
N0	299 (41.0%)	205 (40.2%)	94 (42.7%)	0.461
N1	431 (59.0%)	305 (59.8%)	126 (57.3%)	
**AJCC.M.8th**				
M0	400 (54.8%)	277 (54.3%)	123 (55.9%)	0.105
M1	330 (45.2%)	233 (45.7%)	97 (44.1%)	
**Tumor.size**				
< 6 cm	304 (41.6%)	218 (42.7%)	86 (39.1%)	1
≧ 6 cm	426 (58.4%)	292 (57.3%)	134 (60.9%)	
**Multifocality**				
Multifocal	570 (78.1%)	393 (77.1%)	177 (80.5%)	0.77
Unifocal	160 (21.9%)	117 (22.9%)	43 (19.5%)	
**Reg.LN.Sur**				
None	490 (67.1%)	334 (65.5%)	156 (70.9%)	0.95
1 to 3 removed	89 (12.2%)	67 (13.1%)	22 (10.0%)	
4 or more removed	109 (14.9%)	79 (15.5%)	30 (13.6%)	
Biopsy or aspiration	42 (5.8%)	30 (5.9%)	12 (5.5%)	
**Surgery**				
None surgery	345 (47.3%)	237 (46.5%)	108 (49.1%)	0.705
Lobectomy	138 (18.9%)	94 (18.4%)	44 (20.0%)	
Total/near total thyroidectomy	247 (33.8%)	179 (35.1%)	68 (30.9%)	
**Radiotherapy**				
No	449 (61.5%)	318 (62.4%)	131 (59.5%)	1
Yes	281 (38.5%)	192 (37.6%)	89 (40.5%)	
**Chemotherapy**				
No/Unknown	354 (48.5%)	256 (50.2%)	98 (44.5%)	0.09
Yes	376 (51.5%)	254 (49.8%)	122 (55.5%)	

Note: *Other include American indian/Alaska native, Asian or Pacific islander

**Abbreviations**: **SEER**: Surveillance, Epidemiology, and End Results database; **ATC**: anaplastic thyroid carcinoma; Reg.LN.Sur: Regional lymph node surgery

Except for race (P < 0.001) and no high school diploma (P = 0.006), the clinical characteristics of patients with ATC in the training set and the validation set were not significantly different (P > 0.05). No multicollinearity was found among every variable (VIF<10). Spearman correlation showed that the correlation between no high school population and families below population was strong (0.73), which was low between other variables (all < 0.5) (Supplemental Fig. 1).

### Model results and model performance

**Fig. 2:**
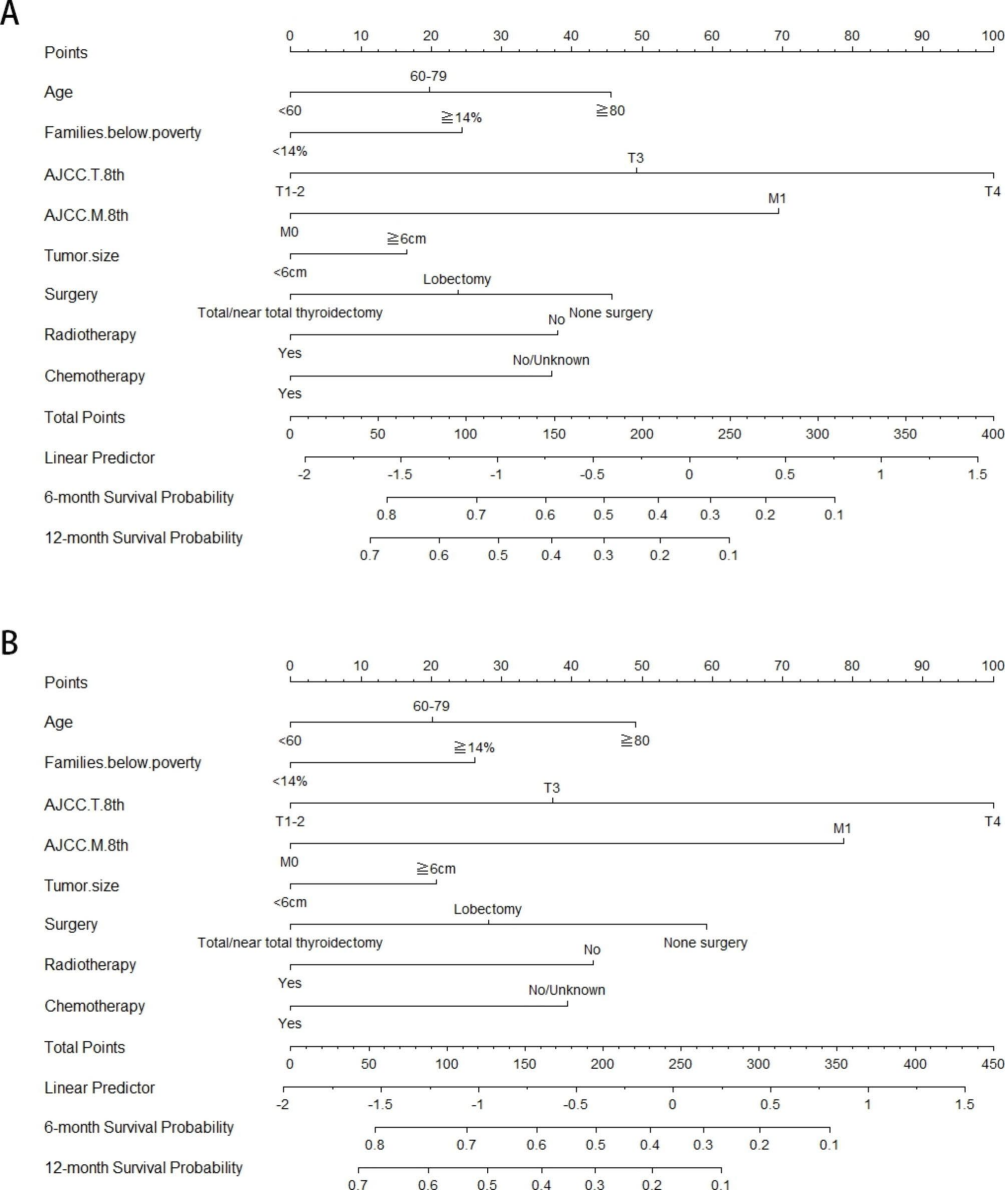
Nomograms for predicting the survival probability of 6-month, 1-year in ATC. Note: (**A**) Predicting 6-month, 1-year OS, (**B**) predicting 6-month, 1-year CSS. (**Abbreviations**: OS: overall survival; CSS: cancer-specific survival)

The results of Cox regression model were displayed in the nomogram (Fig. 2). The total score was obtained by adding the scores corresponding to each predictor. The 6-month and 12-month OS and CSS corresponding to the total score scale under the nomogram were obtained. Age, Families below poverty, AJCC T 8th, AJCC M 8th, tumor size, surgery, radiotherapy, chemotherapy were included in the nomogram for OS and CSS. The tumor stage had the greatest impact on survival, and the therapeutic schedule also affected the survival of patients with ATC: surgery, radiotherapy and chemotherapy. Time-dependent ROC of the nomogram predicting 6-month, 1-year OS and CSS were shown in Supplemental Fig. 2. The C-index values of 6-month, 1-year OS and 6-month, 1-year CSS were 0.782, 0.784, 0.780, 0.777, respectively, indicating acceptable discriminations.

**Fig. 3:**
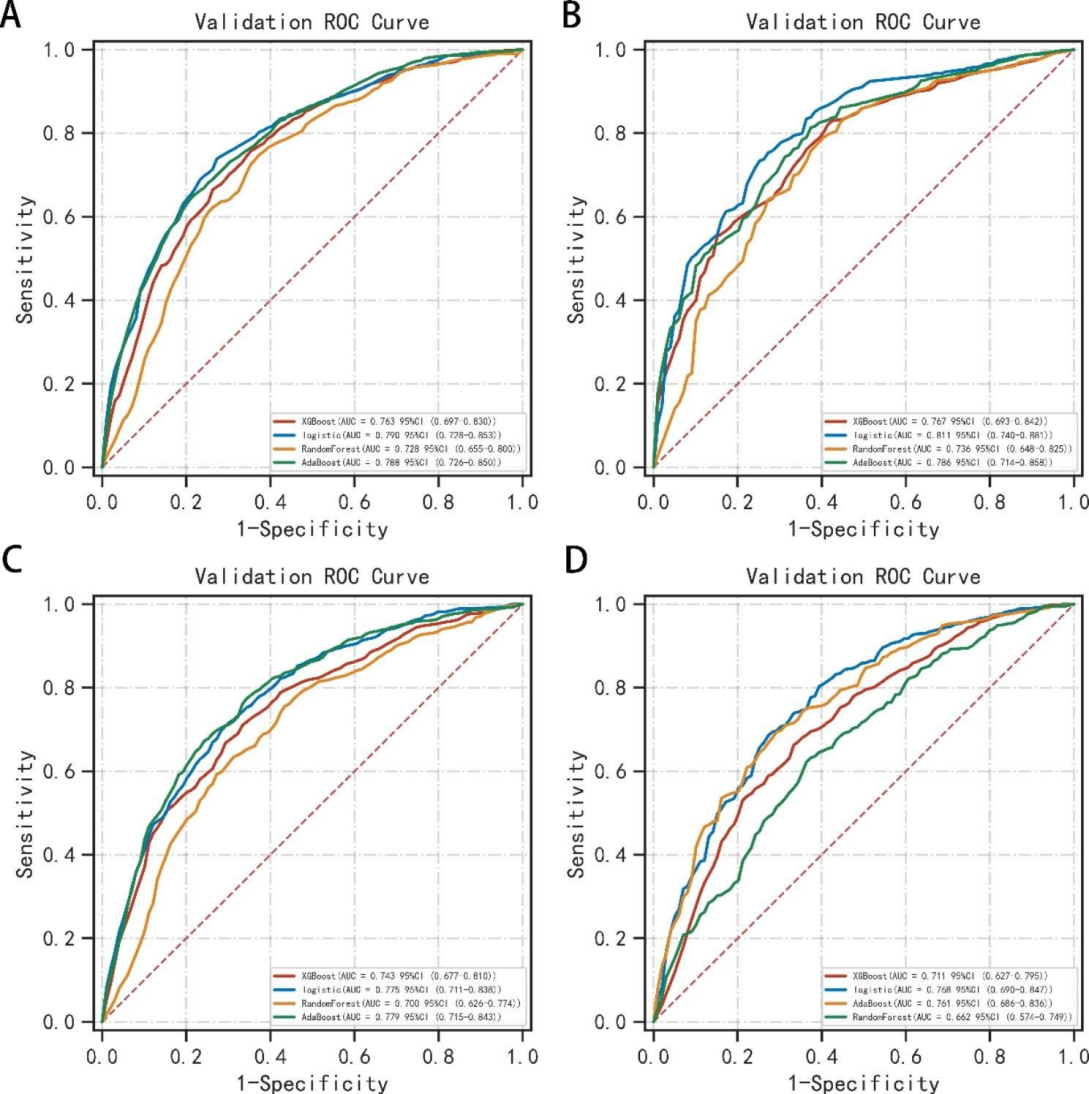
The ROC curves of machine learning models predicting 6-month, 1-year OS and CSS in validation set. (**Note**: (**A**) 6-month OS, (**B**) 12-month OS, (**C**) 12-month OS, (**B**) 12-month CSS. **Abbreviations**: OS: overall survival; CSS: cancer-specific survival; AUC: the area under the receiver operating characteristic curve)

The ROC curves of machine learning algorithms were shown in Fig. 3. C-index values for dichotomous outcomes were summarized in Table 2, including 6-month and 12-month OS and CSS. In the training set, random forest algorithm had the largest C-index value and presented best performance (6-month OS: 0.834; 12-month OS: 0.886; 6-month CSS: 0.857; 12-month CSS: 0.910). In the validation set, the C-index values of random forests algorithm were significantly lower than that in the training set, due to possible overfitting. Combining the results of the training set and the validation set, we found that the logistic algorithm presented best performance. In the validation set, the C-index values of logistic algorithm were (6-month OS:0.790; 12-month OS:0.811; 6-month CSS:0.775; 12-month CSS: 0.768). The results of survival analysis on time-to-event shown that the DeepSurv algorithm presented best performance in the training set and was obviously superior to Cox regression algorithm, OS (DeepSurv: C-index = 0.945, Cox C-index = 0.709), CSS (DeepSurv: C-index = 0.834, Cox: C-index = 0.710). However, in the validation set, the machine learning algorithm did not show superiority over Cox regression, OS (DeepSurv: C-index = 0.658, Cox: C-index = 0.713), CSS (DeepSurv: C-index = 0.834, Cox: C-index = 0.667). In the DeepSurv algorithm, the C-index values of the training set and the validation set were greatly different, and the overfitting was considered.

**Table 2:** Summary of model performance of C-index, Accuracy, Sensitivity, Specificity, Brier-score

	Trainingset	Testset				
	C-index	C-index	Accuracy	Sensitivity	Specificity	Brier score
Classification Models						
**OS 6 month**						
Logistic	0.779	**0.790**	**0.720**	**0.757**	**0.724**	**0.170**
Random Forests	0.834	0.728	0.691	0.737	0.653	0.181
XGBoost	0.808	0.763	0.703	0.696	0.737	0.169
AdaBoost	0.788	0.788	0.699	0.739	0.726	0.238
**OS 12 month**						
Logistic	0.794	**0.811**	**0.728**	**0.829**	**0.677**	**0.117**
Random Forests	0.886	0.736	0.679	0.756	0.659	0.131
XGBoost	0.819	0.767	0.682	0.660	0.778	0.126
AdaBoost	0.803	0.786	0.696	0.784	0.691	0.192
**CSS 6 month**						
Logistic	0.775	**0.775**	**0.700**	**0.770**	**0.679**	**0.179**
Random Forests	0.857	0.700	0.648	0.672	0.689	0.204
XGBoost	0.794	0.743	0.679	0.724	0.677	0.185
AdaBoost	0.774	0.779	0.709	0.772	0.685	0.238
**CSS 12 month**						
Logistic	0.760	**0.768**	**0.721**	**0.735**	**0.704**	**0.127**
Random Forests	0.910	0.662	0.638	0.646	0.639	0.174
XGBoost	0.790	0.711	0.688	0.705	0.653	0.157
AdaBoost	0.775	0.761	0.700	0.680	0.758	0.244
**Time-to-Event Models**						
**OS**						
Cox	**0.709**	**0.713**				
Survival Tree	0.667	0.630				
Survival SVM	0.700	0.651				
Random Survival Forests	0.668	0.630				
XGBoost	0.720	0.657				
DeepSurv	**0.945**	**0.658**				
**CSS**						
Cox	**0.710**	**0.712**				
SurvivalTree	0.670	0.628				
Survival SVM	0.701	0.644				
Random Survival Forests	0.670	0.628				
XGBoost	0.709	0.662				
DeepSurv	**0.834**	**0.676**				

**Abbreviations**: **C-index**: concordance index; **XGBoost**: Extreme Gradient Boosting; **AdaBoost**: Adaptive Boosting; **SVM**: Support Vector Machine; OS: overall survival; CSS: cancer-specific survival

In the machine learning algorithm (logistic, random forest, XGBoost, AdaBoost), we also calculated the accuracy, sensitivity and specificity, as shown in Table 2. The logistic algorithm had high accuracy, sensitivity and specificity, which indicated that the model was effective. Combining values of C-index, accuracy, sensitivity and specificity, we found that logistic algorithm presented best performance in machine learning algorithms. According to the calibration curve, the nomogram predicted the overall survival rate of patients in the training set and the validation set and the actual overall survival rate had good consistency (Supplemental Fig. 3). In addition, the logistic algorithm also shown good consistency in logistic algorithm (Supplemental Fig. 4). As for Brier-score, the value of logistic algorithm was the minimum in machine learning algorithm, indicating the best calibration degree. The Brier-scores of Cox regression and logistic algorithm were similar.

To evaluate the practicability of each model, we plotted the DCA curve (Supplemental Fig. 5 and Supplemental Fig. 6). DCA curve represents the net benefit of clinical decisions. The y-axis represents the net benefit and the x-axis represents the risk threshold. The horizontal line indicates all true negative rates and the diagonal line indicates all true positive rates. This shown that Cox regression model and logistic algorithm had good clinical applicability in predicting the 6-month and 12-month survival rates of ATC and had high net benefits. In the risk stratification KM curves, the patients were divided into high-risk and low-risk groups based on the cut-off values of the total score in the nomogram. For OS, the cut-off value was 169, and for CSS, the cut-off value was 174. As shown in Supplemental Fig. 7, the log rank p was lower than 0.0001, indicating that there was the significant difference between the high-risk group and the low-risk group. The results suggested that the nomogram had high discrimination for the degree of risk.

### Model interpretation of machine learning

**Fig. 4:**
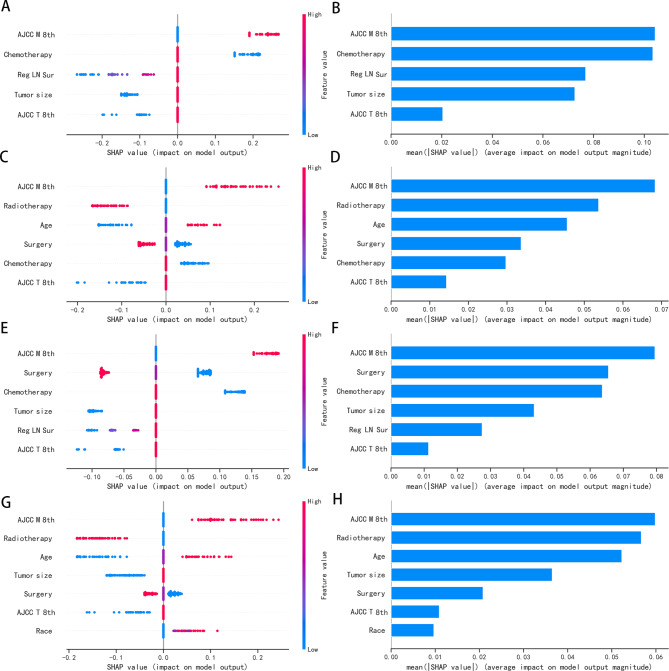
The Logistic model based on the SHAP algorithm. (**Note**: (**A**) The attributes of the features predicting 6-month OS; Y-axis represents features. x-axis represents the degree of influence on the outcome, Each dot represents a sample, the red dots represent the high risk value and the blue dots represent the low risk value。(**B**) Ranking of feature importance predicting 6-month OS; (**C**) The attributes of the features predicting 12-month OS; (**D**) Ranking of feature importance predicting 12-month OS; (**E**) The attributes of the features predicting 6-month CSS; (**F**) Ranking of feature importance predicting 6-month CSS; (**G**) The attributes of the features predicting 12-month CSS; (H) Ranking of feature importance predicting 12-month CSS)

We used SHAP to explain the results of the best machine learning model. Based on the SHAP algorithm, the feature ranking interpretations of the logistic algorithm were shown in Fig. 4. The attributes of the features predicting 6-month OS, 12-month OS, 6-month CSS and 12-month CSS were shown in Fig. 4. In 6-month OS, AJCC M 8th, Chemistry, Regional lymph node surgery, Tumor size and AJCC T 8th were the characteristics of logistic algorithm models, which had the greatest impact on the prediction results. The feature ranking shown that AJCC TNM staging was an important factor for survival prediction of ATC, and AJCC M 8th was the most important feature in OS or CSS.

## Discussion

By comparing the prediction performance of different ML algorithms to the reference method (Cox regression), our findings suggested that Cox regression performed well as a conventional method for ATC survival prediction. Among ML algorithms, Logistic algorithm demonstrated the best performance. Combining SHAP values, Logistic algorithm illustrated key predictive factors and established a high-accuracy survival prediction model. In our study, we used the Cox regression model to identify the most influential predictors and create a nomogram to predict the risk of cancer outcomes for individual patients. The nomogram provides a user-friendly tool for clinicians to assess the risk of cancer outcomes and stratify patients into low- and high-risk groups, which is useful for clinical decision-making. Furthermore, we used the SHAP method to rank the importance of predictors and differentiate their impact on the risk of cancer outcomes. This approach provides a visual and intuitive way to identify protective and risk factors and guide clinical judgment and decision-making.

Our study solved the limitations of ML in predicting the prognosis of ATC survival by including more possible factors. We collected multifaceted disease-related predictors, such as baseline patient information, clinical diagnosis, medical therapy, surgery therapy and so on, we also extracted relevant variables which may influence the development of disease, such as economic condition and education. And the 8th edition of AJCC TNM staging criteria was finally applied to disease strategy for better performance. Our models showed a high C-index value, indicating a remarkable generalization ability and clinical value, providing distinct explanations helping to predict survival rate, which drove clinicians to understand the decision-making process for assessing disease severity.

Different from our study, other researchers tended to apply Cox regression and Logistic regression to analyze risk factors and constructed a predictive model. Gui et al. [13] found that the important predictors for survival rate of ATC were age, historic stage, tumor size, surgery therapy, radiotherapeutic, as analyzed by multivariable Cox proportional hazard regression models. In terms of prediction performance, the nomograms showed a C-indexs value of 0.765 for OS, and 0.773 for CSS. Based on preoperative variables and postoperative variables, Qiu et al. [16] constructed two prognostic nomograms, and the C-index were 0.6783 and 0.7029. The data for the above study were obtained from the SEER database. Meanwhile, a retrospective Study from Regional Registry studied 149 patients with ATC showed that age, tumor size, distant metastasis status were independent variables, definitely affecting survival rate of ATC, as analyzed by multivariable Cox proportional hazard regression [27]. Traditional Cox regression is the most convenient way to solve most survival prediction problems because its results are easy to interpret. However, Cox regression models should be used with a minimum of 10 outcome events per predictor variable (EPV) [28].

ML is an efficient and accurate substitute to semi-parametric and parametric models, with the advantages of high calculating efficiency and excellent performance. ML algorithms do not consider factors of non-proportionality, multicollinearity, or nonlinearity, reducing prediction bias caused by modeling uncertainty. Unfortunately, it’s application in the clinical practice is hindered by the lack of interpretability. Subsequently, SHAP comes into use, aiming to elucidate how the machine models run the output process in an easily understood term, and makes up for the disadvantages mentioned above. There has been no targeted application of machine learning algorithms to predict the survival of patients with anaplastic thyroid carcinoma (ATC). Here, we calculated subject-level survival curves by analyzing outcomes variables in binary model as well as time-event model, providing better understanding of predicted survival. The results of this paper indicated that the models built by ML incorporated fewer predictors and performed no worse than traditional Cox regression. As a substitute of Cox regression, the Logistic algorithm combined with SHAP values performed superiority in clinical applications. However, it is important to note that the predictive efficacy of Cox regression in predicting the survival of ATC patients were comparable with ML algorithms, suggesting that the superiority of ML was not always seen but was seen only in situations when the conventional methods meet their limits.

Deep learning is a branch of machine learning, which requires less data engineering and achieves more accurate prediction when processing a large amount of data. Deep learning has been applied in many fields of medical practice, including image diagnosis, digital pathology, cancer prognosis, etc [29]. Previous studies have shown that the performance of deep learning model in predicting survival analysis is better than that of traditional Cox regression model [30, 31]. We used the deep learning method, named DeepSurv, to predict the survival of ATC patients. The results show that the DeepSurv algorithm is better than Cox regression in the training set. However, no obvious advantages were seen in the validation set. It can be seen that deep learning is challenging in the application of cancer prognosis. The performance of the deep learning model depends on the amount of data [32]. When the amount of patient data is relatively small, sub optimal performance and overfitting problems are usually seen.

Cox regression results showed that Age, Families below poverty, AJCC T 8th, AJCC M 8th, tumor size, surgery, radiotherapy and chemotherapy were important factors in predicting OS and CSS, among which therapeutic approaches were protective factors, including surgery, radiotherapy and chemotherapy. Importantly, older age, higher poverty rate, larger tumor size and more advanced stage suggested a poorer prognosis. Similarly, in the Logistic algorithm analysis, AJCC T 8th and AJCC M 8th were included as important factors in the survival prediction of ATC patients, which was consistent with previous research [33, 34]. By evaluating SHAP values, we found that AJCC M 8th was the most important predictive factor, which is consistent with previous study [13]. In our study, the AJCC.N.8th edition staging was not included into predictive factors. However, regional lymph node surgery was analyzed in the prediction of 6-OS and 6-CSS when using Logistic algorithm. In addition, studies have shown that log odds of positive LN (LODDS) showed better predictive performance than AJCC N states [35]. Radiotherapy and surgery, as compared with control group, improved patient outcomes, being consistent with the findings of Gui et al. [13]. In addition, we found that chemotherapy was also a protective factor for the prognosis of ATC patients.

This study has several limitations. First, this is a retrospective study with small sample size, which may cause bias. More large-scale prospective studies are needed to validate the efficacy of our models. Second, although we included more predictors than previous studies, such as economy, education and marriage, our study did not analyze the impact of immunotherapy and targeted therapy, which were highlighted in recent progress of ATC treatments [23]. Finally, we did not perform performance comparisons with previously established predictive models because of differences in analyzing variables. In the future, we will try to build a deep learning model to predict the prognosis of ATC and conduct hierarchical researches, by analyzing more data and information.

## Conclusion

In conclusion, our study compared the application of Cox regression and ML algorithms in survival prediction of ATC patients. The results of our study showed that Cox regression and Logistic algorithm combined with SHAP value had a good predictive effect in survival prediction of anaplastic thyroid cancer. However, due to the small sample size and lack of external validation, our results need to be viewed more cautiously.

### Electronic supplementary material

Below is the link to the electronic supplementary material.

Supplementary Material 1

Supplementary Material 2

Supplementary Material 3

Supplementary Material 4

Supplementary Material 5

Supplementary Material 6

Supplementary Material 7

## Data Availability

The datasets used and analysed during the current study are available from the SEER database (seer.cancer.gov).

## Abbreviations

ATC: anaplastic thyroid carcinoma
OS: overall survival
CSS: cancer-specific survival
C-index: concordance index
SHAP: SHapley Additive exPlanations
SEER Program: Surveillance, Epidemiology, and End Results database
AJCC: American Joint Committee on Cancer
TNM: Tumor Node Metastasis
ML: machine learning
KNN: K-Nearest Neighbor
VIF: variance inflation factor
XGBoost: extreme gradient boosting
AdaBoost: adaptive boosting
SVM: Survival Support Vector Machine
AIC: Akaike information criterion
DCA curve: Decision Curve Analysis
K-M curve: Kaplan-Meier analysis
