# Correction: Comparison of the cox regression to machine learning in predicting the survival of anaplastic thyroid carcinoma

**DOI:** 10.1186/s12902-023-01431-1

**Published:** 2023-08-15

**Authors:** Lizhen Xu, Liangchun Cai, Zheng Zhu, Gang Chen

**Affiliations:** 1 Shengli Clinical Medical College of Fujian Medical University, Fuzhou, 350001 China; 2 Department of Endocrinology, Fujian Provincial Hospital, Fuzhou, 350000 China; 3 Fujian Provincial Key Laboratory of Medical Analysis, Fujian Academy of Medical Sciences, Fuzhou, China

**Correction:** ***BMC Endocr Disord*23, 129 (2023)**

10.1186/s12902-023-01368-5

Following publication of the original article [1], it was noticed that due to a typesetting error, the copyright holder name was partially missing.

The full copyright holder name should read:

© The Author(s) 2023. **Open Access** This article is licensed under a Creative Commons Attribution 4.0 International License, which permits use, sharing, adaptation, distribution and reproduction in any medium or format, as long as you give appropriate credit to the original author(s) and the source, provide a link to the Creative Commons licence, and indicate if changes were made. The images or other third party material in this article are included in the article’s Creative Commons licence, unless indicated otherwise in a credit line to the material. If material is not included in the article’s Creative Commons licence and your intended use is not permitted by statutory regulation or exceeds the permitted use, you will need to obtain permission directly from the copyright holder. To view a copy of this licence, visit http://creativecommons.org/licenses/by/4.0/. The Creative Commons Public Domain Dedication waiver (http://creativecommons.org/publicdomain/zero/1.0/) applies to the data made available in this article, unless otherwise stated in a credit line to the data.

The original article [1] has been updated.
