# Supplementary figures and images for: Comparison of the cox regression to machine learning in predicting the survival of anaplastic thyroid carcinoma
Source: BMC Endocr Disord. 2023 Jun 5;23:129. doi: 10.1186/s12902-023-01368-5 (PMC10249166; doi:10.1186/s12902-023-01368-5)

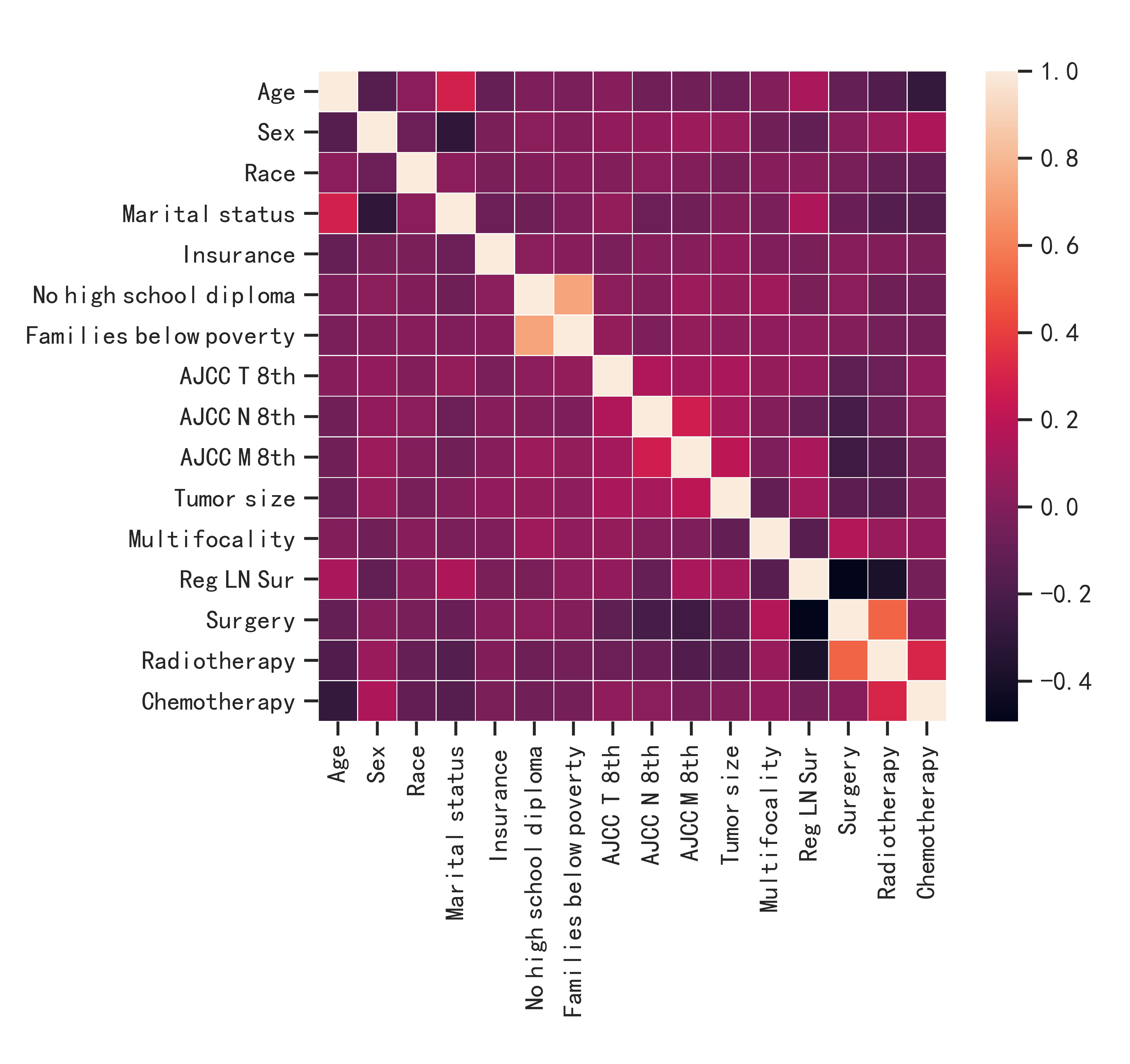

Supplement: Supplementary file 1 — Supplementary Material 1 [file 12902_2023_1368_MOESM1_ESM.tif]

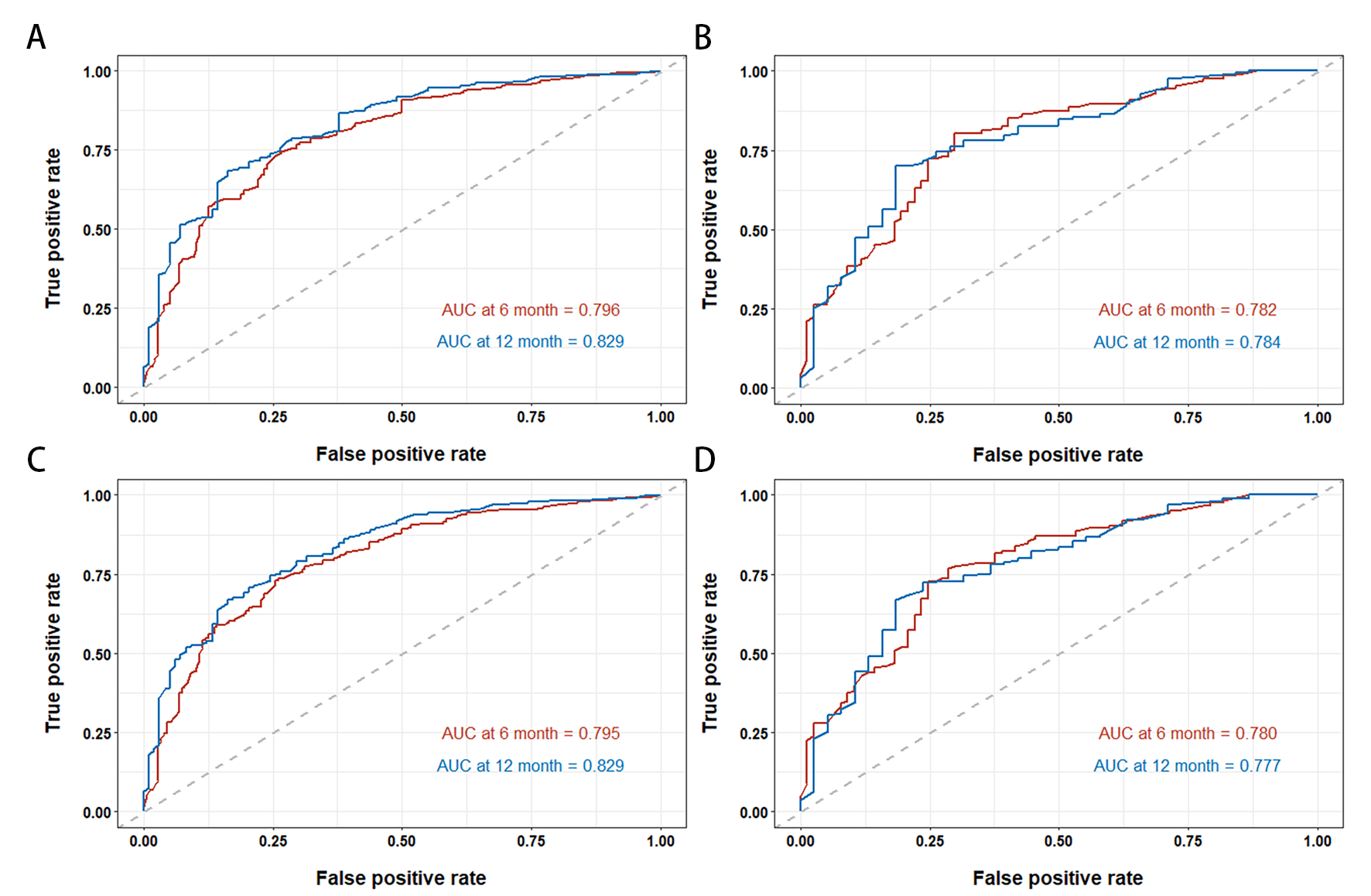

Supplement: Supplementary file 2 — Supplementary Material 2 [file 12902_2023_1368_MOESM2_ESM.tif]

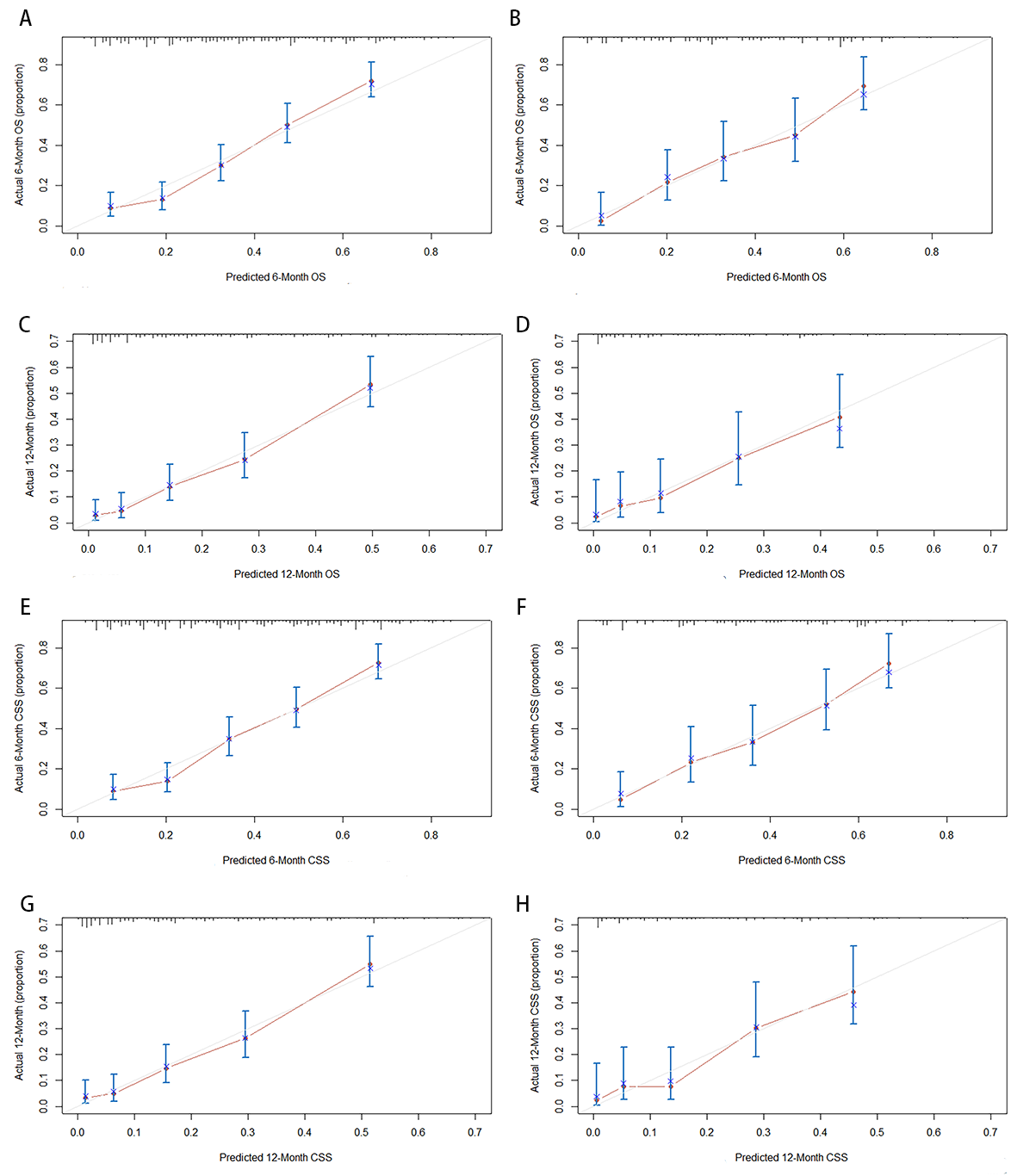

Supplement: Supplementary file 3 — Supplementary Material 3 [file 12902_2023_1368_MOESM3_ESM.tif]

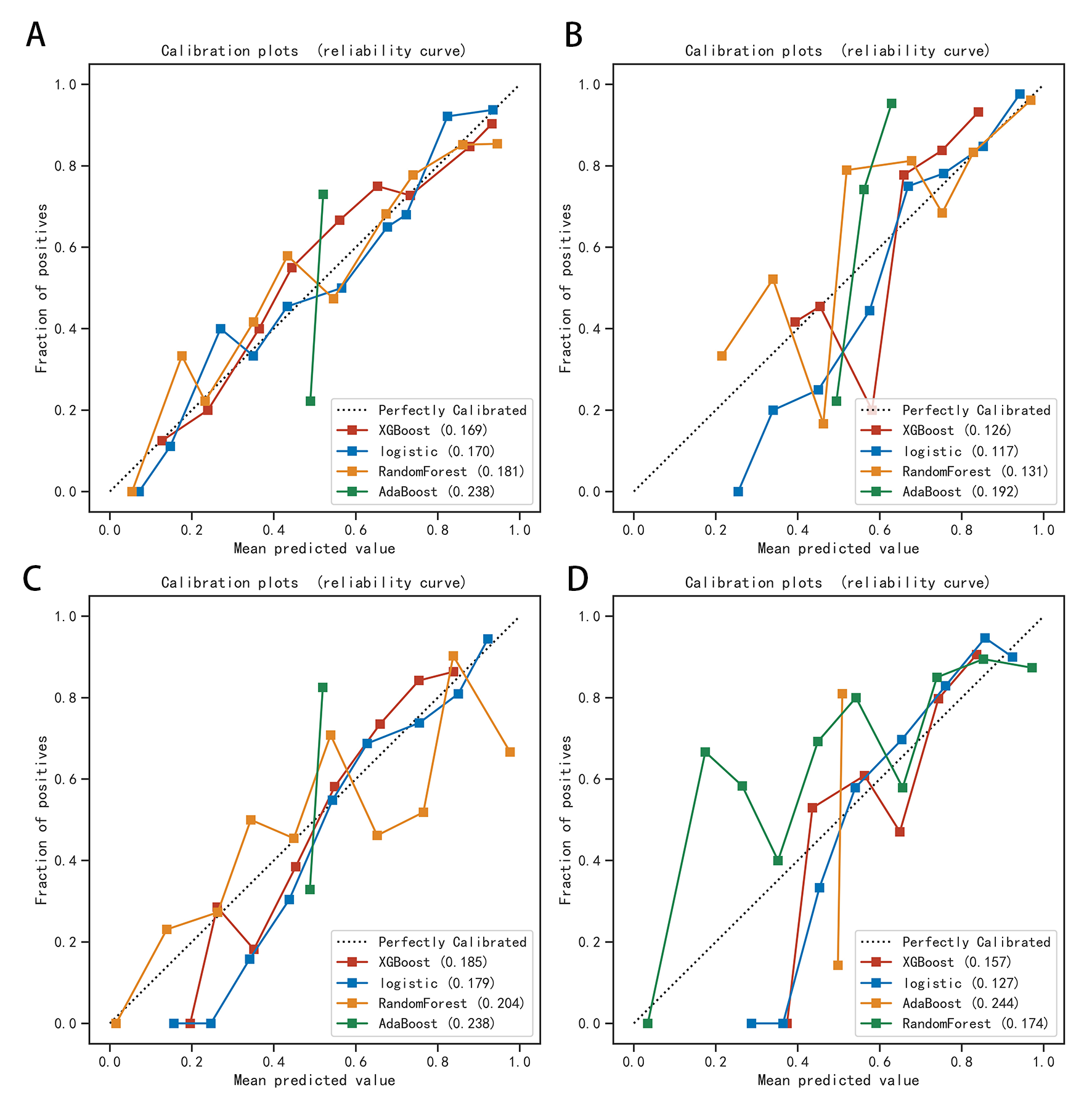

Supplement: Supplementary file 4 — Supplementary Material 4 [file 12902_2023_1368_MOESM4_ESM.tif]

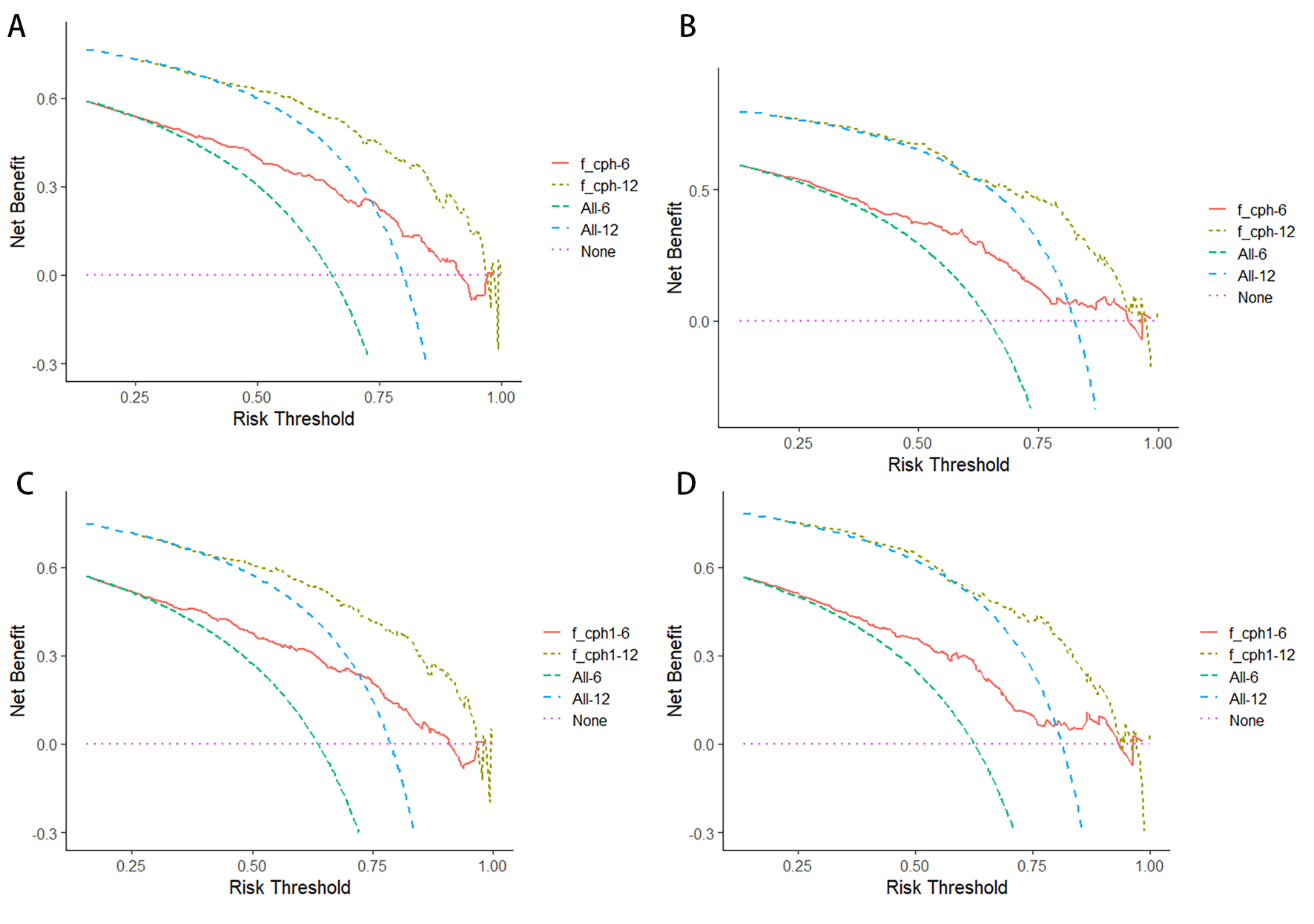

Supplement: Supplementary file 5 — Supplementary Material 5 [file 12902_2023_1368_MOESM5_ESM.tif]

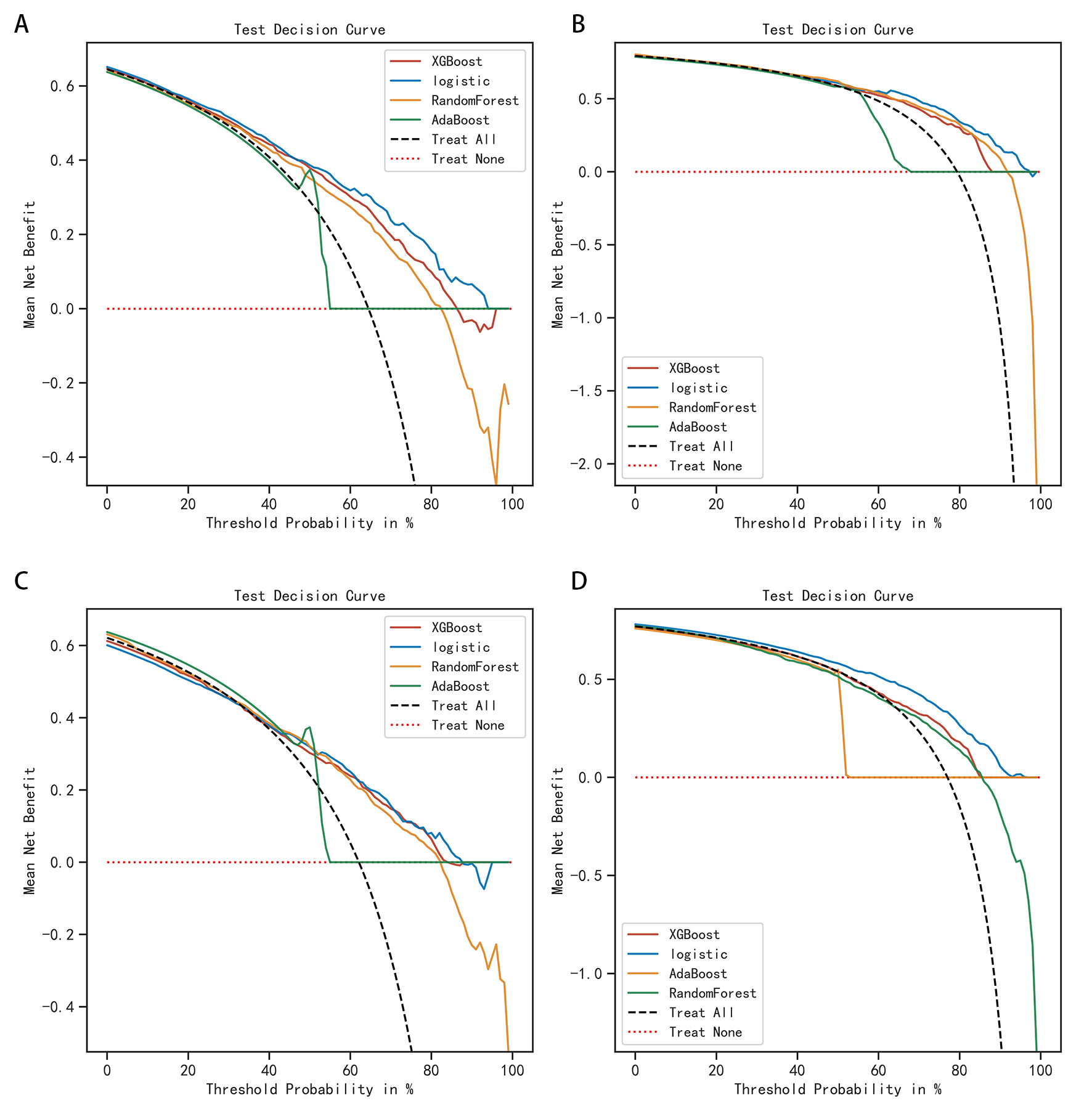

Supplement: Supplementary file 6 — Supplementary Material 6 [file 12902_2023_1368_MOESM6_ESM.tif]

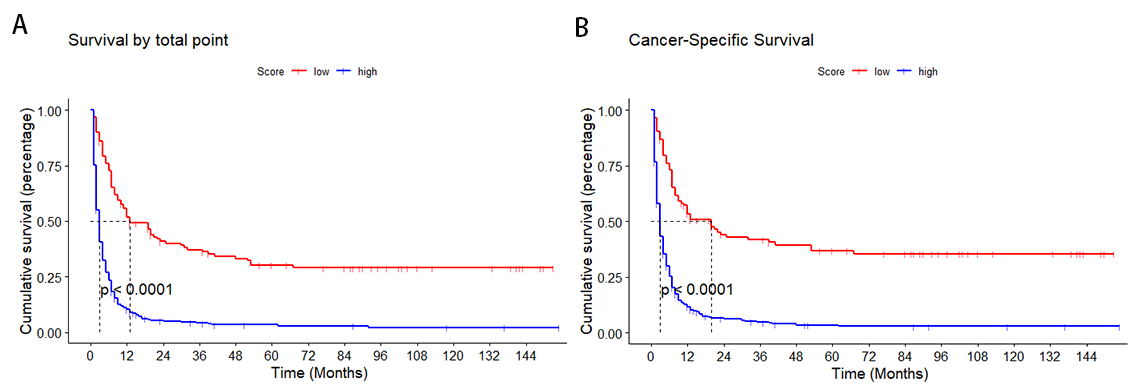

Supplement: Supplementary file 7 — Supplementary Material 7 [file 12902_2023_1368_MOESM7_ESM.tif]
